# Cardiotoxicity of immune checkpoint inhibitors: A frequency network meta-analysis

**DOI:** 10.3389/fimmu.2022.1006860

**Published:** 2022-09-14

**Authors:** Maobai Liu, Xitong Cheng, Ruping Ni, Bin Zheng, Shunmin Huang, Jing Yang

**Affiliations:** ^1^ Department of Pharmacy, Fujian Medical University Union Hospital, Fuzhou, China; ^2^ College of Pharmacy, Fujian Medical University, Fuzhou, China

**Keywords:** cancer, cardiovascular adverse events, myocarditis, immune checkpoint inhibitors, network meta-analysis

## Abstract

Immune checkpoint inhibitors (ICIs) in combination withother anti-cancer treatments have been approved for a variety of cancers. While the difference in the incidence of cardiovascular adverse events has not been fully investigated. We aimed to assess the the differences in cardiotoxicity among cancer patients receiving different ICI therapies. PubMed, Embase, Web of Science, Cochrane Library, and ClinicalTrials.gov. websites were searched for all randomized controlled trials (RCTs) of ICI. The primary outcomes were any grade cardiotoxicity and Grade 3-5 cardiotoxicity, the secondary outcomes were any grade myocarditis and Grade 3-5 myocarditis, with sub-analyses based on cancer type and does of ICI. A systematic review and frequency network meta-analysis were then performed for cardiotoxicity events. 91 RCTs (n=52247) involving 12 treatment arms were finally included. We observed that PD-L1 + CTLA-4 had the highest risk among all therapies inducing any grade cardiotoxicity, and the differences were significant except PD-1 + CTLA-4, PD-1 + TTD and PD-L1 + TTD. In addition, CTLA-4 had a higher risk of Grade 3-5 cardiotoxicity than PD-1 and anit-PD-L1. For Grade 1-5 myocarditis and Grade 3-5 myocarditis, no significant difference was found among differences therapies. No differences were observed in subgroup analyses according to does and cancer type. There were differences in the incidence of cardiotoxicity among different ICI therapies. For ICI monotherapy, CTLA-4 may be linked to Grade 3-5 cardiotoxicity than PD-1 or PD-L1. For dual therapy, the cardiotoxicity of dual ICI therapy seems to be higher than that of chemotherapy or targeted therapy.

## Introduction

Recently, immune checkpoint inhibitors (ICIs) have greatly improved the outcomes of several cancers and have become the hotspot of cancer research (1). ICIs include anti-cytotoxic T-lymphocyte antigen-4 (CTLA-4), anti-programmed cell death 1 (PD-1), and anti-programmed cell death ligand 1 (PD-L1). Immune checkpoint inhibitors induce CD8-positive T cells to kill cancer cells and enhance the anti-tumor response of the immune system by blocking the inhibitory signal of T cell activation (2, 3). However, they may cause damage to normal tissues while attacking tumor cells and leading to the occurrence of immune-related adverse events (irAEs).

These irAEs can affect any organ system. The common irAEs include diarrhea, colitis, hepatitis, skin toxicities and endocrinopathies, with fewer irAEs in cardiovascular, hematologic, renal, neurologic and ophthalmologic (4). Although cardiotoxicity are uncommon, they are serious irAEs which may cause the interruption, termination of immunotherapy or even the death of the patient. Common cardiotoxicity include coronary artery disease, heart failure, myocarditis, atrial fibrillation, and pericardial disease. The mortality of myocarditis is up to 39.7% - 50%, ranking first among all irAEs (5–9).

Currently, ICIs are often used in combination with chemotherapeutic drugs or targeted therapeutic drugs (10). And more and more evidence supports the clinical value of combining appropriately chemotherapies or targeted therapies with ICI (11, 12). However, the effects of the combination therapy on cardiotoxicity are not clear. Due to its relatively low incidence, it is often easily ignored by clinicians. Although there is no strong evidence from clinical trials, early surveillance and identification of cardiotoxicity, timely discontinuation of ICI, and the use of corticosteroid therapy are essential to improve the prognosis (13). Therefore, this study conducted a network meta-analysis to compare the risk of cardiotoxicity with different combinations of ICI therapies and to provide a reference for the safety of clinical treatment with ICI.

## Methods

### Study objectives

The incidence and mortality of cardiotoxicity of different ICI therapies were analyzed. The primary objective was to compare the risk of Grade 1-5 cardiotoxicity and Grade 3-5 cardiotoxicity associated with different ICI therapies. The secondary objective was to compare the risk of Grade 1-5 myocarditis and Grade 3-5 myocarditis associated with different ICI therapies. Subgroup analyses were performed to evaluate the impact of tumour type and doses of ICI.

### Search strategy and data extraction

This network meta-analysis was conducted according to PRISMA guidelines (14). The RCTs comparing all ICI (alone or in combination with standard therapy) with other non-ICI therapy or single-agent ICI with double-agent ICI was comprehensively searched in PubMed, Embase, Web of Science, Cochrane Library and ClincalTrials.gov from January 1, 2014 to January 1, 2022. The keywords included “PD‐1”, “PD-L1”, “immune checkpoint inhibitors”, “atezolizumab”, “avelumab”, “durvalumab”, “ipilimumab”, “nivolumab”, “pembrolizumab”, “camrelizumab”, “tremelimumab”, “toripalimab”, “sintilimab”, “cancer” (**Supplementary Table 1**).

The inclusion criteria for this study were as follows (1): Patients: those with cancer whether they received basic treatment or not (2); Interventions: the treatment group received ICI, either alone or in combination. The control group received placebo, non-ICI based therapy or different doses of the same ICI (3); Study type: randomized controlled trials, the language was limited to English (4); Outcomes: we used common terminology criteria for adverse events (CTCAE) version 5 to determine cardiotoxicity. We excluded (1): single-arm trials (2); studies with incomplete data or without full text available.

Two researchers (XTC and RPN) independently extracted the data and evaluated the quality of the included studies. If there was any disagreement, a consensus should be reached after consultation with a third reviewer (JY). For each eligible study, the extracted data included (1): study information (first author, study name, publication year, National Clinical Trial number) (2); patient baseline information (sample size, median age, follow-up, tumor type, lines of treatment) (3); treatment group and control group (treatments in each arm, type of ICI, cardiovascular adverse events in each arm).

### Risk of bias assessment

Risk of bias assessment was undertaken by two independent investigators (XTC and RPN) using RevMan5.4 software, according to the RCT bias risk assessment tool recommended by the Cochrane Collaboration Handbook (15).

### Statistical analysis

Stata version17.0 software was used. Freeman Tukey double inverse sine transform method was used to calculate the incidence and mortality of cardiotoxicity. We used risk ratios (RRs) and 95% confidence intervals (95% CI) as summary statistics to quantify the risk of cardiotoxicity. The network meta-analysis was undertaken by the Frequency model (16). The data were fitted with an inconsistent model and a consistent model respectively to test the overall consistency. The node splitting method and ring inconsistency test were utilized to test the local consistency. If the P-value > 0.05, it indicated that there was no inconsistency, and the consistency model could be employed for analysis (17). The surface under the cumulative ranking (SUCRA) curve was calculated to rank the cardiotoxicity with different regimens. The smaller the SUCRA, the greater the risk of cardiotoxicity and the lower the safety (18). The publication bias and small sample effect were detected by Begg’ test.

## Results

### Study characteristics

**Figure 1:**
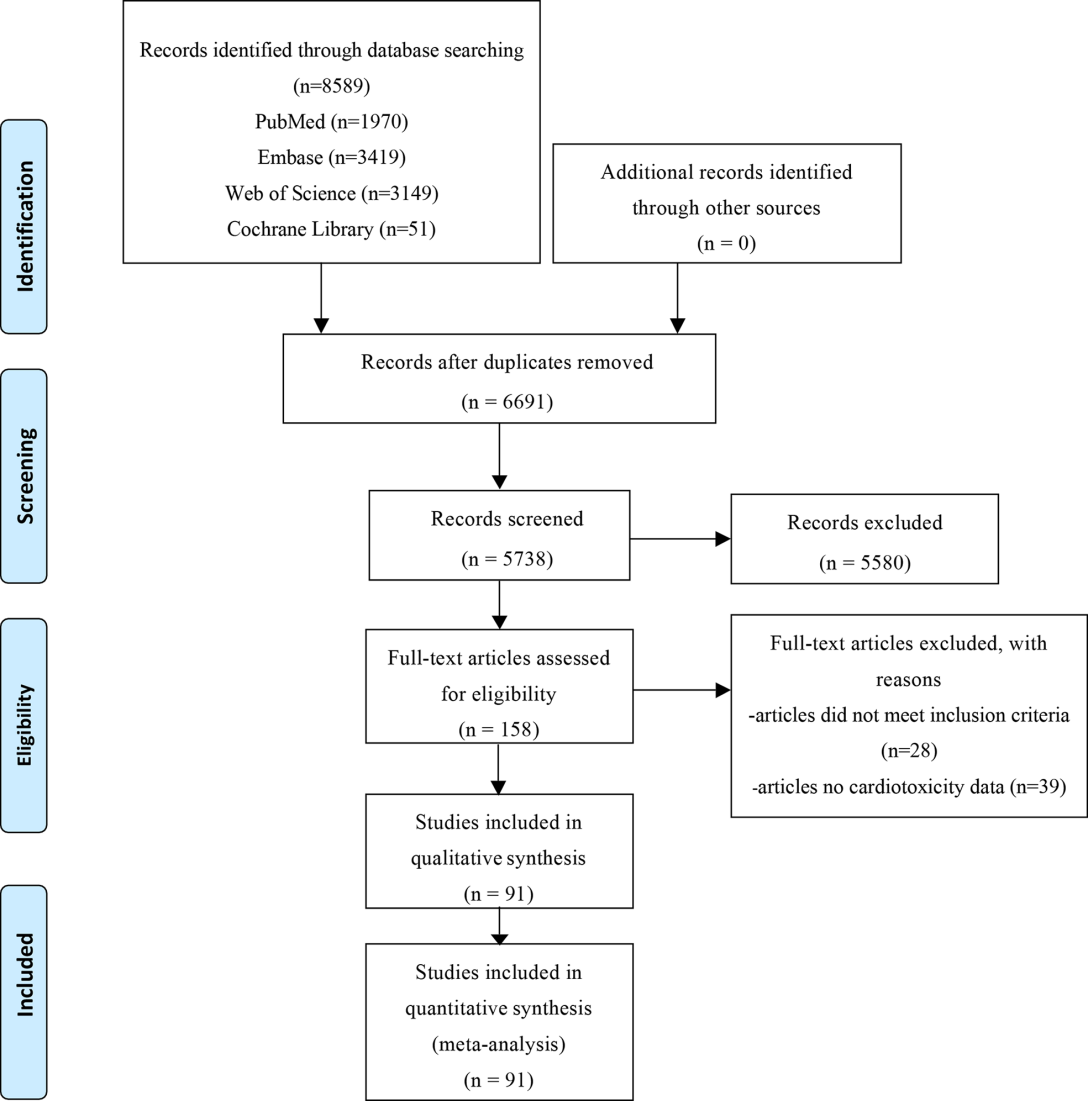
PRISMA flowchart of retrieved studies.

**Figure 2:**
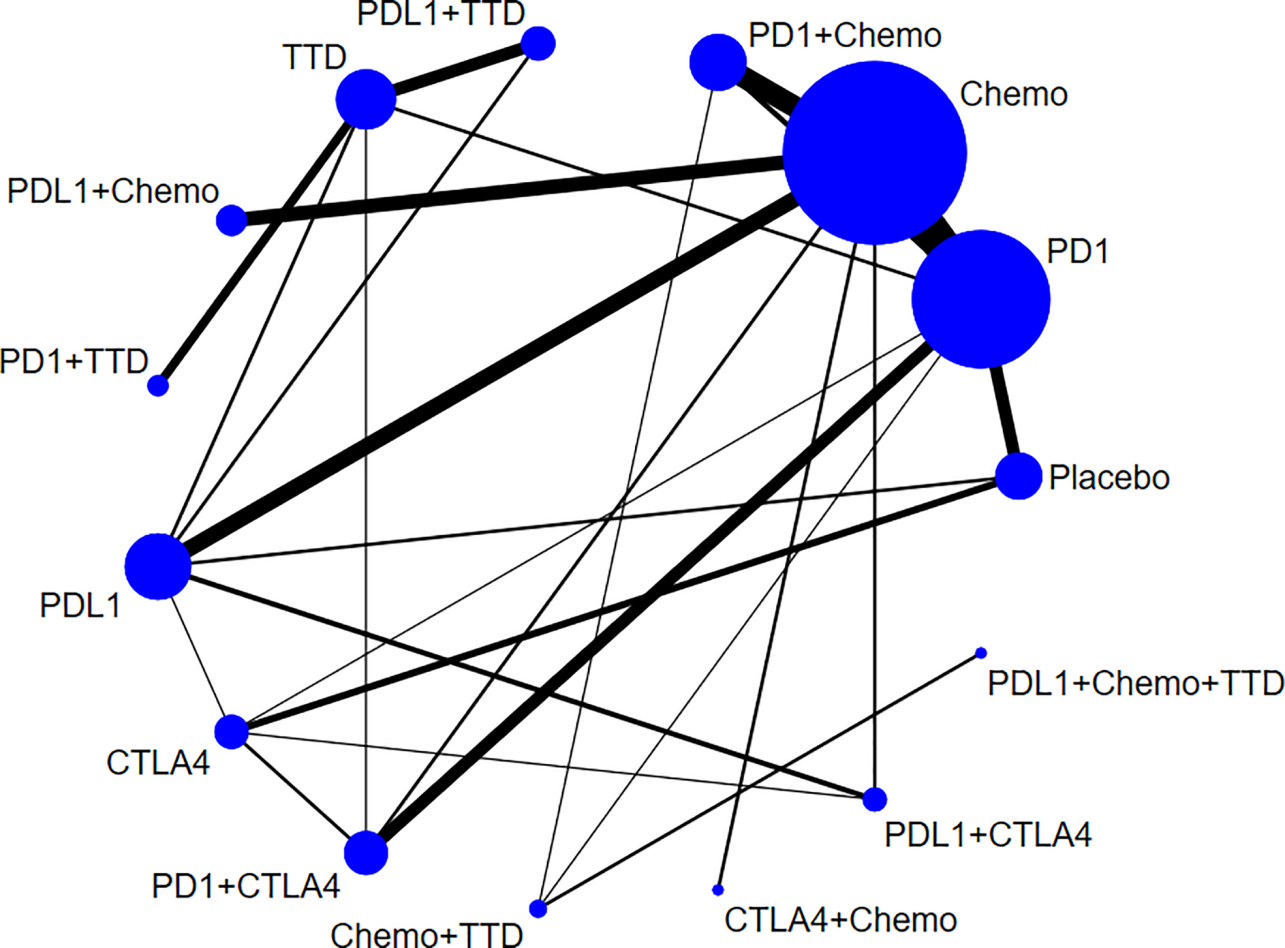
Network plots for Grade 1-5 cardiotoxicity. The area of the circle represents the sample size of the treatment measure, the line represents the study and the line thickness represents the number of studies.

8,589 related literatures were obtained after a comprehensive search. Finally, 91 studies (52,247 subjects) were included, after reading 158 potential studies (**Figure 1**). The characteristics of the included studies is show in **Supplementary Table 2**. All RCTs reported Grade 1-5 cardiotoxicity. 75 RCTs reported Grade 3-5 cardiotoxicity. 36 RCTs reported Grade 1-5 myocarditis, and 24 RCTs reported Grade 3-5 myocarditis. The included studies involved 17 cancer types, of which lung cancer accounted for 33%. Among the 91 articles, 58% compared ICI to chemotherapy. The network plot of Grade 1-5 cardiotoxicity is shown in **Figure 2**.

### Risk of bias assessment and publication bias

Assess risk bias according to the Cochrane Collaboration Handbook, and all included studies had a low risk of bias. We utilized the Begg’s test to evaluate the publication bias. The results are as follows: Grade 1-5 cardiotoxicity (*P*=0.696)>0.05, Grade 3-5 cardiotoxicity (*P*=0.547)>0.05, Grade 1-5 myocarditis (*P*=0.139)>0.05, and Grade 3-5 myocarditis (*P*=0.088)>0.05. The scatter distribution on the Begg funnel is basically symmetrical, suggesting that there was no significant publication bias of the included studies (**Supplementary Figure 1**).

### Incidence of cardiotoxicity

**Figure 3:**
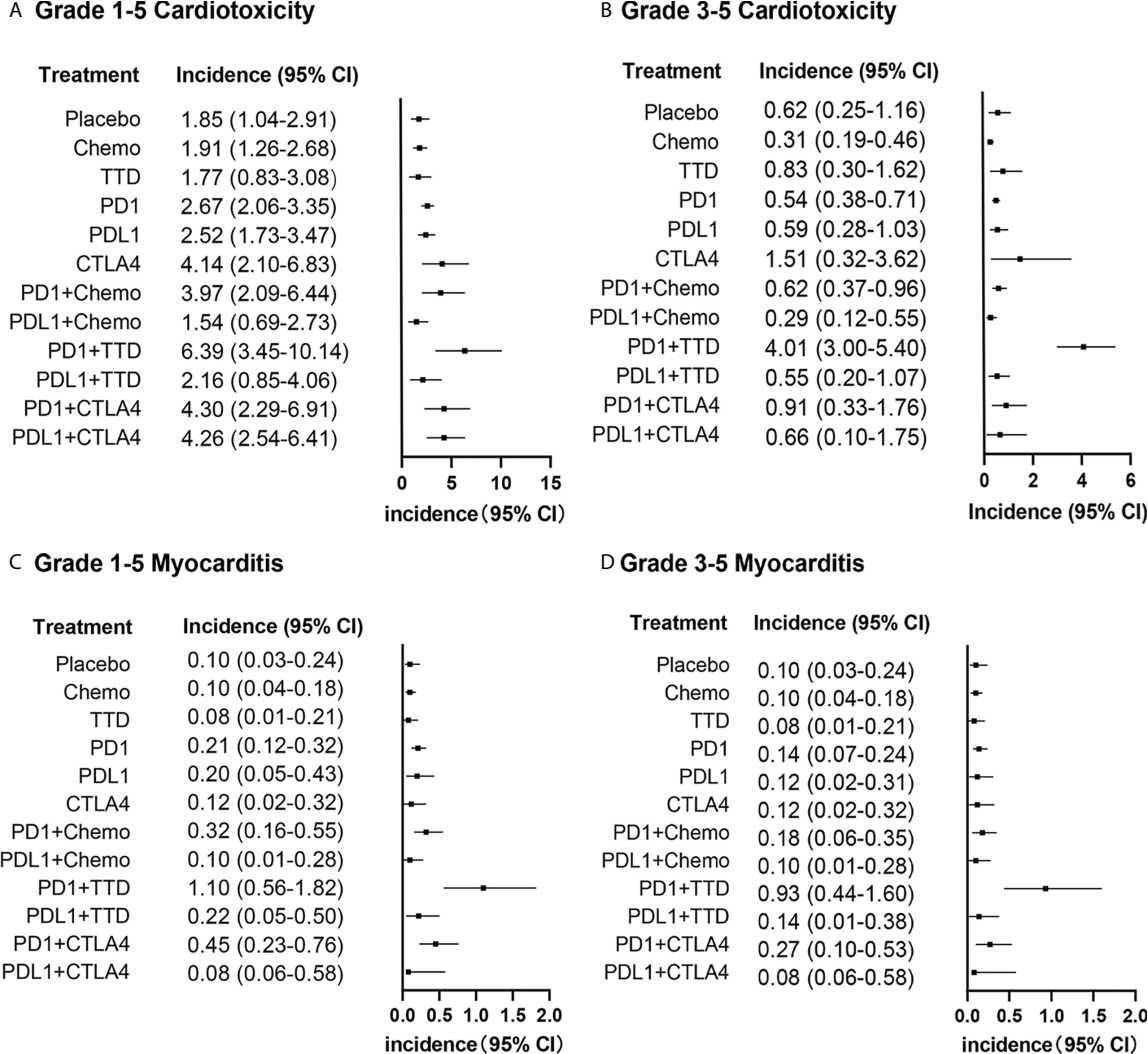
Incidence of cardiotoxicity among different therapies. **(A)**: Incidence of Grade 1-5 cardiotoxicity among different therapies, **(B)**: Incidence of Grade 3-5 cardiotoxicity among different therapies, **(C)**: Incidence of Grade 1-5 myocarditis among different therapies, **(D)**: Incidence of Grade 3-5 myocarditis among different therapies.

The incidence of Grade 1-5 cardiotoxicity, Grade 3-5 cardiotoxicity, Grade 1-5 myocarditis and Grade 3-5 myocarditis were 3.23% (839/25998), 0.97% (220/22718), 0.35% (43/12270) and 0.33% (25/7528), respectively. Different therapies had separate incidences of cardiotoxicity. The incidence of Grade 1-5 cardiotoxicity, Grade 3-5 cardiotoxicity, Grade 1-5 myocarditis and Grade 3-5 myocarditis with PD-1 + Chemo, PD-1 + TTD and PD-1 + CTLA-4 were relatively high. The highest incidences of Grade 1-5 cardiotoxicity, Grade 3-5 cardiotoxicity, Grade 1-5 myocarditis and Grade 3-5 myocarditis were from PD-1 + TTD, up to 6.39% (95% CI: 3.45-10.14), 4.10% (95% CI: 3.00-5.40), 1.10% (95% CI: 0.56-1.82) and 0.93% (95% CI: 0.44-1.60) respectively (**Figure 3**).

### Fatal cardiotoxicity

A total of 66 fatal cardiovascular adverse events occurred (17 cardiac arrest, 12 myocardial infarction, 12 cardiac failure, 8 myocarditis, 17 others). The combined mortality was calculated by using the random effect model Freeman Tukey double inverse sine conversion. The total mortality of cardiotoxicity with ICI was 0.55% (95% CI: 0.40%-0.73%). The highest mortality of cardiotoxicity with PD-1 + TTD was 1.84% (95% CI: 0.30%-4.68%), followed by PD-1 + CTLA-4 1.55% (95% CI: 0.06%-5.02%). There was no significant difference between ICI therapy and non-ICI therapy (RR=1.35, 95% CI: 0.91-2.01) (**Table 1**).

**Table 1:** Mortality and risk ratio of cardiotoxicity by treatment regimens.

ICIs type				Mortality				RR	95% CI	P
		number of events / sample size		treatment group		control group				
	N	Treatment group	control group	%	95% CI	%	95% CI			
Total	39	66/12620	36/10890	0.55**^a^**	0.40-0.73	0.36^b^	0.25-0.48	1.35**^c^**	0.91-2.01	1.00
PD-1 vs. Placebo	4	5/1137	2/715	0.59	0.23-1.12	0.42	0.08-1.03	1.23	0.30-5.06	0.89
PD-1 vs. Chemo	9	10/3013	5/2944	0.45	0.24-0.72	0.25	0.10-0.46	1.57	0.59-4.16	0.99
PD-1+Chemo vs. Chemo	4	11/1172	5/836	0.85	0.48-1.34	0.58	0.24-1.06	1.25	0.42-3.68	0.54
PD-1+CTLA-4 vs. Chemo	2	2/681	0/670	0.43	0.08-1.06	0.07	0.01-0.42	2.94	0.31-28.16	0.97
PD-1+CTLA-4 vs. TTD	1	0/547	3/535	0.05	0.04-0.40	0.65	0.15-1.52	0.14	0.01-2.70	–
PD-1+CTLA-4 vs. CTLA-4	1	1/94	0/46	1.55	0.06-5.02	0.53	0.48-4.60	1.48	0.06-35.74	–
PD-1+CTLA-4 vs. PD-1	2	1/362	1/362	0.74	0.29-5.00	0.47	0.03-1.43	1.02	0.11-9.75	0.33
PD-1+TTD vs. TTD	4	18/1071	10/1053	1.84	0.30-4.68	0.96	0.28-2.05	1.59	0.73-3.44	0.51
PD-L1 vs. Chemo	2	2/739	1/711	0.40	0.07-0.98	0.21	0.01-0.68	1.54	0.19-12.46	0.59
PD-L1+CTLA4 vs. PD-L1	1	2/371	1/369	0.66	0.10-1.75	0.40	0.01-1.28	1.99	0.18-21.84	–
PD-L1+Chemo vs. Chemo	5	5/1821	7/1348	0.32	0.11-0.65	0.58	0.20-1.17	0.49	0.14-1.71	0.71
PD-L1+TTD vs. TTD	1	1/434	0/439	0.34	0.01-1.10	0.06	0.05-0.50	3.03	0.12-74.28	–
CTLA-4 vs. Placebo	3	8/1178	1/862	0.75	0.25-1.54	0.17	0.003-0.61	2.84	0.61-13.22	0.98

N: number of head to head comparison groups, Heterogeneity test:a: Q=61.58 I^2 =^ 36.7% P=0.012, b: Q=43.27 I2 = 11.2% P=0.271, c: Q=16.82 I2 = 0.0% P=1.00.

### Conventional pairwise meta-analysis

**Figure 4:**
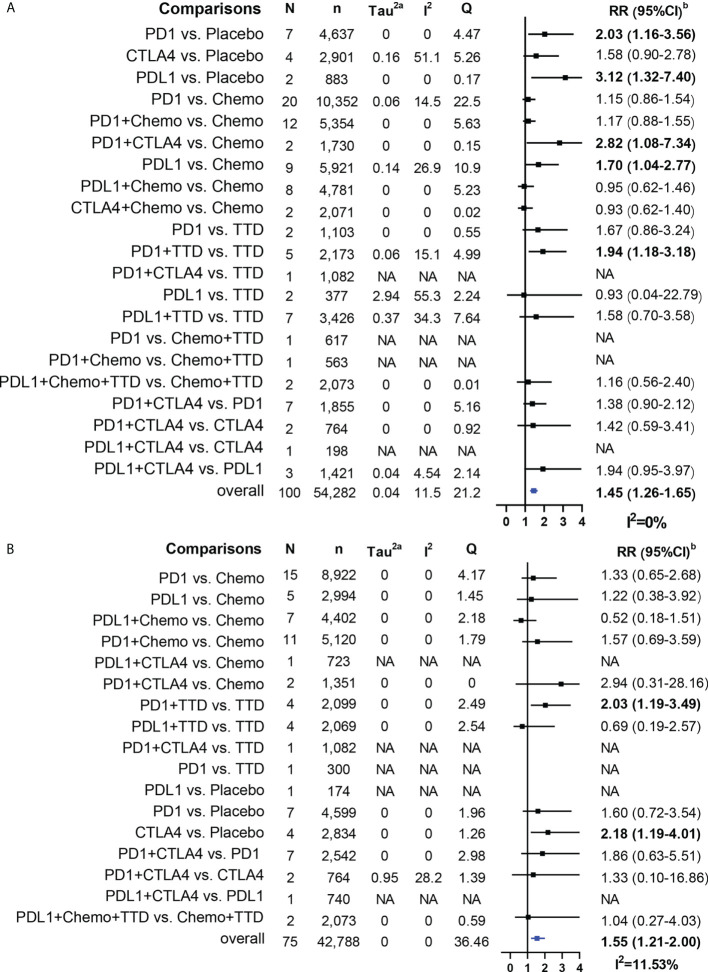
Results of traditional pairwise meta-analysis for Grade 1-5 cardiotoxicity and Grade 3-5 cardiotoxicity. **(A)**: Forestplot for Grade 1-5 cardiotoxicity, **(B)**: Forestplot for Grade 3-5 cardiotoxicity, N: mumber of head to head comparison groups, NA, no available, a, Tau^2^ represents between-study heterogeneity characterized by standard deviation; b, Bold values denote statistical significance.

**Figure 5:**
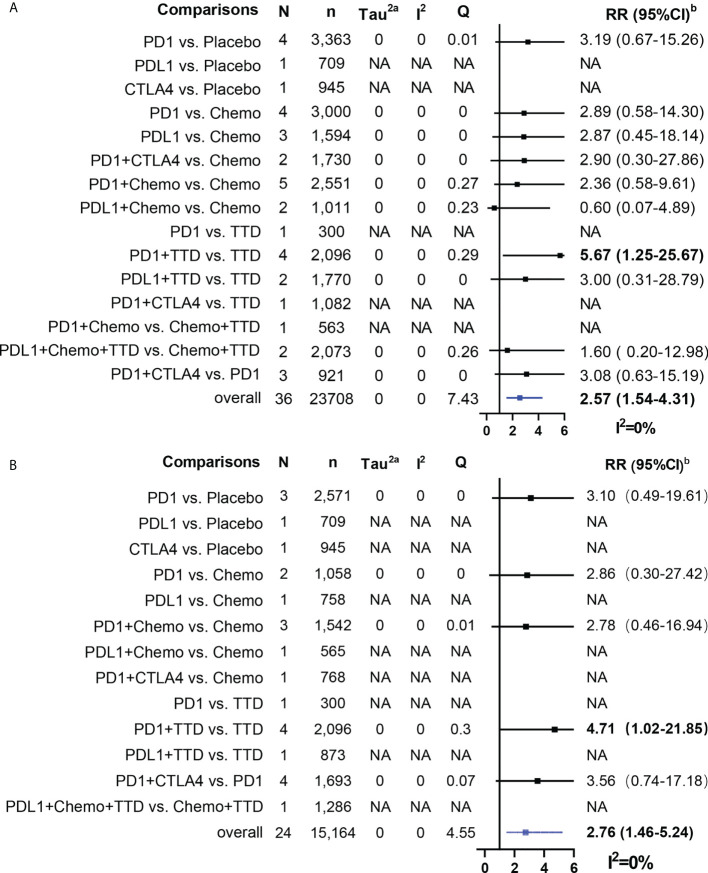
Results of traditional pairwise meta-analysis for Grade 1-5 myocarditis and Grade 3-5 myocarditis. **(A)**: Forestplot for Grade 1-5 myocarditis, **(B)**: Forestplot for Grade 3-5 myocarditis, N: mumber of head to head comparison groups, NA, no available; a, Tau^2^ represents between-study heterogeneity characterized by standard deviation; b, Bold values denote statistical significance.

Overall, ICI treatment increased the risk of cardiotoxicity compared with non-ICI treatment (Grade 1-5 cardiotoxicity: RR = 1.45, 95% CI: 1.26-1.65; Grade 3-5 cardiotoxicity: RR = 1.55, 95% CI: 1.21-2.00; Grade 1-5 myocarditis: RR = 2.58, 95% CI: 1.54-4.31; Grade 3-5 myocarditis: RR = 2.76, 95% CI: 1.45-5.24; **Figures 4**, **5**). For Grade 1-5 cardiotoxicity, PD-1 (RR=2.03, 95% CI: 1.16-3.56) and PD-L1 (RR=3.12, 95% CI: 3.12-7.40) were statistically significant compared with placebo, but CTLA-4 showed no significant difference. PD-L1 (RR=1.70, 95% CI: 1.04-2.77) and PD-1+ CTLA-4 (RR=2.82, 95% CI: 1.08-7.34) were statistically significant compared with chemotherapy. PD-1 + TTD (RR=1.94, 95% CI: 1.18-3.18) was statistically significant compared with TTD. For Grade 3-5 cardiotoxicity, there was no significant difference between PD-1 and PD-L1 compared with placebo, whereas CTLA-4 (RR=2.34, 95% CI: 1.26-4.36) was statistically significant. PD-1 + TTD (RR=2.20, 95% CI: 1.29-3.75) was statistically significant compared with TTD. There was no significant difference between the other group treated with ICI and the control group. For Grade 1-5 myocarditis and Grade 3-5 myocarditis, only PD-1 + TTD vs. TTD was statistically significant (RR=5.67, 95% CI: 1.25-25.67; RR=4.71, 95% CI: 1.02-21.85).

In the sensitivity analysis, the pooled RR estimates were stable, with only minimal fluctuations when excluding each study sequentially.

### Network meta-analysis

**Figure 6:**
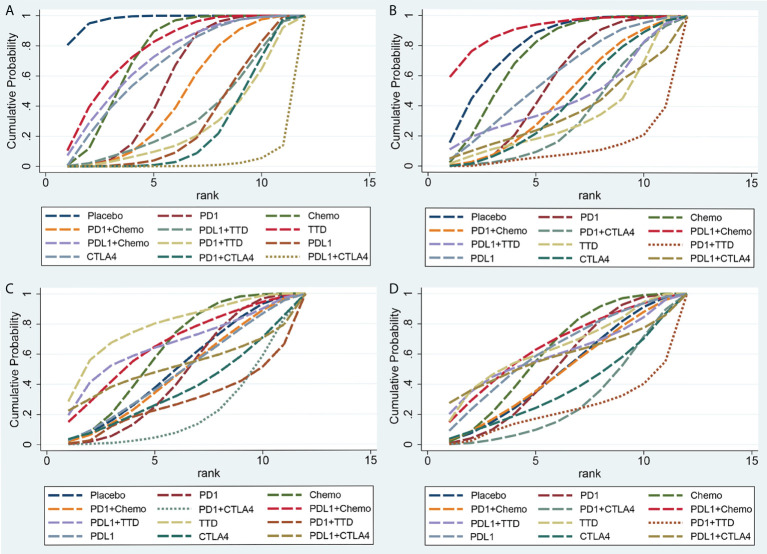
Rankings of SUCRA for the risk of cardiotoxicity. **(A)** Grade 1-5 cardiotoxicity, **(B)** Grade 3-5 cardiotoxicity, **(C)** Grade 1-5 myocarditis, **(D)** Grade 3-5 myocarditis. SUCRA, surface under the cumulative ranking.

We ranked the probabilities of cardiovascular adverse events for all treatments by estimating the SUCRA value (**Figure 6**). Detailed probabilities of cardiovascular adverse events are showed in **Supplementary Figure 2**. A lower SUCRA value indicated a higher probability of cardiotoxicity. For Grade 1-5 cardiotoxicity, the corresponding ranking of incidences of the twelve groups from high to low was: PD-L1 + CTLA-4 (2.1%), PD-1 + CTLA-4 (22.6%), PD-1 + TTD (25.6%), PD-L1 (29.3%), PD-L1+TTD (33.2%), PD-1+Chemo (46.4%), PD-1 (55.2%), CTLA-4 (66.9%), PD-L1+Chemo (71.2%), Chemo (73.3%), TTD (76.5%), Placebo (97.5%). For Grade 3-5 cardiotoxicity, the corresponding ranking of incidences of the twelve groups from high to low was: PD-1+TTD (11.1%), PD-1+CTLA-4 (33.0%), TTD (33.1%), PD-L1+CTLA-4 (35.4%), CTLA-4 (41.5%), PD-1+Chemo (45.0%), PD-L1+TTD (45.0%), PD-1 (54.3%), PD-L1(58.2%), Chemo (72.5%), Placebo (80.1%), PD-L1+Chemo (90.9%). For Grade 1-5 myocarditis, the corresponding ranking of incidences of the twelve groups from high to low was: PD-1+CTLA-4 (20.9%), PD-1+TTD (28.3%), CTLA-4 (37.0%), PD-1 (45.4%), PD-1+Chemo (46.7%), PD-L1 (47.5%), Placebo (49.7%), PD-L1+CTLA-4 (52.6%), Chemo (61.8%), PD-L1+Chemo (65.7%), PD-L1+TTD (65.8%), TTD (78.4%). For Grade 3-5 myocarditis, the corresponding ranking of incidences of the twelve groups from high to low was: PD-1+TTD (21.9%), PD-1+CTLA-4 (27.4%), CTLA-4 (36.8%), PD-1+Chemo (47.4%), Placebo (48.0%), PD-1 (51.0%), PD-L1+CTLA-4 (55.7%), PD-L1+TTD (60.0%), Chemo (60.4%), PD-L1 (61.4%), TTD (64.4%), PD-L1+Chemo (65.6%).

There was not any significant difference in terms of the risk of Grade 1-5 cardiotoxicity between different ICI monotherapy. In addition, the risk of Grade 1-5 cardiotoxicity in ICI monotherapy was not higher than that in single chemotherapy or single targeted therapy, except PD-L1 vs. Chemo (RR=1.53, 95% CI: 1.06-2.20). According to the ranking of SUCRA, PD-L1 + CTLA-4 ranked first among all therapies inducing Grade 1-5 cardiotoxicity, and the differences were significant except PD-1 + CTLA-4, PD-1 + TTD and PD-L1 + TTD (PD-1: RR=0.35, 95% CI: 0.17-0.72; PD-L1: RR=0.48, 95% CI: 0.26-0.89; CTLA-4: RR=3.19, 95% CI: 1.46-6.96; PD-1+Chemo: RR=0.42, 95% CI: 0.20-0.87; PD-L1 + TTD: RR=2.54, 95% CI: 1.15-5.61; TTD: RR=3.52, 95% CI: 1.54-8.05; Chemo: RR=3.19, 95% CI: 1.61-6.32; **Table 2**). PD-1 + CTLA-4 ranked first for Grade 1-5 myocarditis, PD-1 + TTD ranked first for Grade 3-5 cardiotoxicity and Grade 3-5 myocarditis, but there was no significant difference between other therapies except PD-1 + TTD vs. TTD (**Table 3**).

**Table 2:** Network estimates of treatment comparisons for Grade 1-5 cardiotoxicity (on the lower triangle) and Grade 3-5 cardiotoxicity (on the upper triangle).

PD-1	1.64 (0.65,4.16)	1.19 (0.47,3.03)	4.52 (0.46,44.76)	0.93 (0.28,3.02)	1.87 (0.23,15.18)	0.37 (0.11,1.30)	1.54 (0.12,20.47)	1.30 (0.53,3.22)	0.71 (0.37,1.36)	2.23 (0.24,20.73)	0.60 (0.29,1.25)
**0.69 (0.48,0.99)**	**PD-1+CTLA-4**	0.73 (0.20,2.59)	2.76 (0.28,26.88)	0.56 (0.13,2.41)	1.14 (0.12,10.90)	0.23 (0.05,1.03)	0.94 (0.07,12.31)	0.79 (0.25,2.57)	0.44 (0.15,1.27)	1.36 (0.15,12.44)	0.37 (0.12,1.10)
0.84 (0.62,1.15)	1.22 (0.77,1.93)	**PD-1+Chemo**	3.80 (0.32,44.56)	0.78 (0.21,2.84)	1.57 (0.18,13.52)	0.31 (0.08,1.16)	1.29 (0.08,20.01)	1.10 (0.31,3.92)	0.60 (0.28,1.28)	1.88 (0.17,20.73)	0.51 (0.16,1.61)
0.68 (0.34,1.36)	0.98 (0.49,1.96)	0.80 (0.38,1.69)	**PD-1+TTD**	0.20 (0.02,2.66)	0.41 (0.02,9.10)	0.08 (0.01,1.10)	0.34 (0.08,1.41)	0.29 (0.03,3.28)	0.16 (0.01,1.68)	**0.49 (0.29,0.85)**	0.13 (0.01,1.44)
0.74 (0.49,1.11)	1.07 (0.64,1.79)	0.88 (0.56,1.37)	1.09 (0.51,2.34)	**PD-L1**	2.02 (0.31,13.15)	0.40 (0.09,1.81)	1.66 (0.10,28.19)	1.41 (0.35,5.70)	0.77 (0.27,2.23)	2.41 (0.20,29.57)	0.65 (0.18,2.36)
**0.35 (0.17,0.72)**	0.51 (0.24,1.10)	**0.42 (0.20,0.87)**	0.52 (0.21,1.33)	**0.48 (0.26,0.89)**	**PD-L1+CTLA-4**	0.20 (0.02,1.95)	0.82 (0.03,22.69)	0.70 (0.07,6.51)	0.38 (0.05,2.87)	1.19 (0.06,25.07)	0.32 (0.04,2.81)
1.15 (0.69,1.89)	1.66 (0.90,3.05)	1.36 (0.80,2.30)	1.69 (0.72,3.98)	1.55 (0.87,2.76)	**3.24 (1.42,7.38)**	**PD-L1+Chemo**	4.14 (0.24,72.27)	3.51 (0.77,15.95)	1.92 (0.66,5.58)	6.01 (0.47,76.08)	1.62 (0.39,6.70)
0.75 (0.37,1.50)	1.08 (0.53,2.20)	0.89 (0.42,1.87)	1.10 (0.55,2.20)	1.01 (0.48,2.14)	2.11 (0.83,5.39)	0.65 (0.28,1.52)	**PD-L1+TTD**	0.85 (0.06,12.73)	0.46 (0.03,6.58)	1.45 (0.39,5.40)	0.39 (0.03,5.64)
1.13 (0.67,1.89)	1.64 (0.94,2.86)	1.34 (0.75,2.39)	1.67 (0.74,3.76)	1.53 (0.85,2.74)	**3.19 (1.46,6.96)**	0.99 (0.49,2.00)	1.51 (0.66,3.45)	**CTLA-4**	0.55 (0.19,1.60)	1.71 (0.16,18.32)	**0.46 (0.26,0.83)**
1.13 (0.90,1.42)	**1.64 (1.09,2.46)**	**1.34 (1.02,1.76)**	1.67 (0.81,3.41)	**1.53 (1.06,2.20)**	**3.19 (1.61,6.32)**	0.99 (0.63,1.54)	1.51 (0.74,3.08)	1.00 (0.58,1.72)	**Chemo**	3.13 (0.31,31.31)	0.84 (0.33,2.15)
1.24 (0.75,2.07)	**1.80 (1.07,3.03)**	1.48 (0.83,2.63)	**1.84 (1.17,2.89)**	1.69 (0.92,3.09)	**3.52 (1.54,8.05)**	1.09 (0.54,2.19)	1.66 (0.98,2.83)	1.10 (0.56,2.18)	1.10 (0.64,1.89)	**TTD**	0.27 (0.03,2.75)
**1.84 (1.18,2.85)**	**2.66 (1.57,4.51)**	**2.18 (1.30,3.66)**	**2.71 (1.21,6.06)**	**2.49 (1.48,4.17)**	**5.19 (2.42,11.15)**	1.60 (0.84,3.06)	**2.46 (1.10,5.47)**	**1.63 (1.12,2.37)**	**1.63 (1.02,2.60)**	1.48 (0.77,2.82)	**Placebo**

The summary estimates are risk ratios (RRs) and 95% confidence intervals. For Grade 1-5 cardiovascular AEs, the column-defining treatment is compared to the row-defining treatment, and RRs < 1 favor the column-defining treatment. For Grade 3-5 cardiovascular AEs, the row-defining treatment is compared to the column-defining treatment, and RRs > 1 favor the row-defining treatment. Bold values denote statistical significance.

**Table 3:** Network estimates of treatment comparisons for Grade 1-5 myocarditis (on the lower triangle) and Grade 3-5 myocarditis (on the upper triangle).

PD-1	1.77(0.59,5.33)	1.07 (0.30,3.80)	3.02(0.16,57.45)	0.75 (0.16,3.61)	0.76 (0.02,26.48)	0.67 (0.13,3.46)	0.70 (0.03,14.30)	1.51 (0.25,9.00)	0.82 (0.34,1.96)	0.64 (0.05,7.96)	1.08 (0.34,3.46)
0.53 (0.18,1.54)	**PD-1+CTLA-4**	0.60 (0.12,3.00)	1.71(0.09,33.79)	0.42 (0.07,2.66)	0.43(0.01,16.97)	0.38 (0.06,2.54)	0.39 (0.02,8.40)	0.85 (0.13,5.42)	0.46 (0.13,1.69)	0.36 (0.03,4.71)	0.61 (0.14,2.71)
0.88 (0.28,2.80)	1.67 (0.37,7.48)	**PD-1+Chemo**	2.83 (0.12,68.81)	0.70 (0.12,4.11)	0.71 (0.02,26.52)	0.63 (0.11,3.56)	0.66 (0.03,17.04)	1.41 (0.17,11.98)	0.76 (0.27,2.17)	0.60 (0.04,9.91)	1.01 (0.19,5.29)
0.52(0.03,7.79)	0.98 (0.06,14.95)	0.59 (0.03,11.02)	**PD-1+TTD**	0.25 (0.01,6.87)	0.25 (0.00,25.03)	0.22 (0.01,6.36)	0.23(0.02,2.24)	0.50 (0.02,14.76)	0.27 (0.01,5.73)	**0.21 (0.05,0.99)**	0.36 (0.02,8.28)
1.00 (0.23,4.45)	1.90 (0.33,10.98)	1.14 (0.22,5.85)	1.94 (0.09,42.03)	**PD-L1**	1.01(0.03,32.54)	0.90 (0.12,6.65)	0.93 (0.03,27.52)	2.01 (0.22,18.19)	1.09 (0.26,4.63)	0.86 (0.05,16.32)	1.43 (0.27,7.64)
1.17 (0.03,40.38)	2.23 (0.06,86.26)	1.34 (0.04,48.06)	2.28 (0.03,193.72)	1.17 (0.04,37.26)	**PD-L1+CTLA-4**	0.88 (0.02,37.02)	0.92 (0.01,95.92)	1.98 (0.04,98.49)	1.07(0.03,34.48)	0.85 (0.01,64.80)	1.42 (0.04,54.42)
1.58 (0.31,8.03)	3.00 (0.46,19.44)	1.80 (0.34,9.66)	3.07 (0.13,70.98)	1.58 (0.23,10.97)	1.35 (0.03,56.01)	**PD-L1+Chemo**	1.04 (0.03,31.74)	2.24 (0.21,23.95)	1.22 (0.30,4.85)	0.96 (0.05,18.92)	1.60 (0.23,11.20)
2.05 (0.13,32.64)	3.90 (0.24,62.65)	2.34 (0.12,46.00)	3.99 (0.44,35.91)	2.05 (0.09,46.41)	1.75 (0.02,153.70)	1.30 (0.05,31.34)	**PD-L1+TTD**	2.15 (0.07,67.88)	1.17 (0.05,26.55)	0.92 (0.17,4.89)	1.54 (0.06,38.29)
0.74 (0.13,4.37)	1.41 (0.22,8.84)	0.85 (0.11,6.68)	1.44 (0.06,34.43)	0.74 (0.09,6.42)	0.63 (0.01,31.13)	0.47 (0.04,4.91)	0.36 (0.01,9.02)	**CTLA-4**	0.54 (0.08,3.71)	0.43 (0.02,8.77)	0.71 (0.14,3.55)
1.30 (0.56,3.03)	2.46 (0.70,8.62)	1.48 (0.57,3.82)	2.52 (0.15,42.19)	1.30(0.33,5.03)	1.11 (0.03,35.17)	0.82 (0.21,3.27)	0.63 (0.04,11.11)	1.75 (0.26,11.64)	**Chemo**	0.79 (0.06,11.07)	1.32 (0.34,5.17)
2.91 (0.30,27.81)	5.53 (0.57,53.52)	3.31 (0.27,40.95)	**5.65 (1.25,25.55)**	2.91 (0.20,42.33)	2.48 (0.04,162.15)	1.84 (0.12,28.89)	1.42 (0.29,7.01)	3.92 (0.24,63.96)	2.24 (0.21,24.26)	**TTD**	1.67 (0.11,26.04)
1.08 (0.35,3.31)	2.04 (0.48,8.75)	1.22 (0.26,5.79)	2.09 (0.11,38.47)	1.07 (0.21,5.40)	0.92 (0.02,34.80)	0.68 (0.10,4.60)	0.52 (0.03,10.12)	1.45 (0.29,7.17)	0.83 (0.22,3.10)	0.37 (0.03,4.47)	**Placebo**

The summary estimates are risk ratios (RRs) and 95% confidence intervals. For Grade 1-5 myocarditis, the column-defining treatment is compared to the row-defining treatment, and RRs < 1 favor the column-defining treatment. For Grade 3-5 myocarditis, the row-defining treatment is compared to the column-defining treatment, and RRs > 1 favor the row-defining treatment. Bold values denote statistical significance.

The subgroup analysis for Grade 1-5 cardiotoxicity by cancer types and does of ICI. There was no significant difference in cardiotoxicity with different therapies for different cancer types (**Supplementary Table 3**). In the subgroup analysis based on dose (**Supplementary Figure 3**), compared with targeted therapy, Atezolizumab 1200 mg (q3w) treatment regimen increased the risk of cardiotoxicity (RR = 2.39, 95% CI: 1.04-5.49), while Atezolizumab 840 mg (q2w) regimen did not (RR = 0.51, 95% CI: 0.55-5.68). Through indirect comparison, there is no significant difference between Atezolizumab 1200 mg (q3w) and 840 mg (q2w) (RR = 0.44, 95% CI: 0.15-1.32).

In the point splitting method and ring inconsistency test (**Supplementary Figure 4**), the P values of the four outcome indicators were greater than 0.05, suggesting that there was no inconsistency in the whole and local.

## Discussion

In our systematic review and network meta-analysis, we analyzed the cardiotoxicity risk of different cancer treatment regimens and the effects of different cancer types or different doses of ICI on cardiotoxicity. Currently, whether ICI therapies will increase the risk of cardiotoxicity remains controversial. Some studies have shown that ICI treatment is not associated with a higher risk of cardiotoxicity (19, 20). However, this result may be due to the low incidence of ICI related cardiotoxicity and the limited analysis data included. In our study, we performed a comprehensive analysis of 91 RCTs (including 52247 patients and 12 treatment arms) and found that the use of ICI was associated with a higher risk of cardiotoxicity compared with non-ICI treatments. At present, the mechanism of immune related cardiotoxicity is unclear, but studies have shown that ICI blocking CTLA-4, PD-1 and PD-L1 signaling pathways may be one of the causes of autoimmune heart disease (21, 22).

For comparison among single ICI therapies, studies have shown that the incidence of high-grade toxicity in patients with metastatic melanoma receiving ipilimumab 10mg/kg is 57.9%. In contrast, PD-1 or PD-L1 caused high-grade adverse events in only 10%-15% of patients (23, 24). In our study, it was also found that CTLA-4 seems to be associated with Grade 3-5 cardiotoxicity compared with PD-1 and PD-L1, but the mechanism remains unclear.

For comparison of dual ICI therapy with single therapy, the risk of cardiotoxicity was higher with dual ICI therapy than with chemotherapy, targeted therapy and single ICI therapy. Based on the evidence network, the risk of cardiotoxicity with PD-L1 + CTLA-4 was higher than that with PD-1,PD-L1 and CTLA-4. The risk of cardiotoxicity of PD-1 + CTLA-4 is only higher than that with PD-1. Although PD-1 + CTLA-4 vs. PD-1, PD-L1 + CTLA-4 vs. PD-L1 and PD-L1 + CTLA-4 vs. CTLA-4 are not statistically significant in the traditional meta-analysis, this may be due to few included studies in this comparison groups and subsequently wide confidence intervals. According to the consistency of network meta-analysis, the results of network meta-analysis still have credibility (25).

For comparison among dual therapies, except that the risk of cardiotoxicity of PD-L1 + CTLA-4 was significantly higher than that of PD-L1 + Chemo, there was no significant difference in the risk of cardiotoxicity between dual ICI therapy and single ICI combined with chemotherapy or targeted therapy. Studies have shown that the incidence of cardiotoxicity is higher with dual ICI therapy than the incidence treated with ICI monotherapy, either as single treatment or in combination with chemotherapy (26). The explanation of the increased cardiotoxicity of dual ICI therapy likely obeys the different signaling pathways inhibited by PD-1 and CTLA-4 molecules on T Cells (27). On the one hand, it prevents the binding of CTLA-4 and B7 molecules. On the other hand, it blocks the inhibitory signal produced by the binding of PD-1 with PD-L1 (28, 29).

Although this study did not find significant differences in the risk of myocarditis between different ICI treatments, special attention should be paid to dual ICI therapy. It has been reported that the risk of myocarditis in patients treated with ICI therapy increases dramatically and dual ICI therapy has a higher incidence of myocarditis, with more serious symptoms and higher mortality (30). The mechanism of ICI-related myocarditis is unclear, but several preclinical studies have made some assumptions, such as inflammation caused by over activation of T cells (21, 22). The specific mechanism behind it needs to be further studied.

Of the 52,247 treated patients, 66 cardiovascular-related fatal events were reported. Cardiovascular iRAEs occur with an incidence of 1.14%–5%, but have the highest mortality rate among iRAEs up to 50% (31). The mortality of ICI-related cardiotoxicity cannot be ignored. In our study, there was no significant difference in the mortality of cardiotoxicity between the groups treated with ICI and the control group. The mortality of cardiotoxicity with PD-1 + TTD was the highest (1.84%). However, due to the rarity of cardiovascular-related fatal events with ICI and the difference in the number of therapies included in the study, this conclusion was uncertain. Although we did not find that the risk of cardiotoxicity was related to cancer type and dose of ICI. Previous studies indicated that the occurrence of irAEs induced by PD-1/PD-L1 agents is related to cancer types and is dose-independent (32, 33). So far, the mechanism underlying this result has not been well elucidated.

Based on the current network evidence, we suggest that patients at high risk of cardiotoxicity (such as pre-existing cardiovascular conditions, previous and concomitant cardiotoxic treatments) with monotherapy choose PD-1/PD-L1 or appropriately reduce the dose of CTLA-4 within the effective dose range. For patients with no response to monotherapy or poor cardiac function, it is recommended to give priority to single ICI combined with chemotherapy.

There are two limitations in the network meta-analysis of this study. One limitation is that the incidence of cardiotoxicity with ICI is low. The trials included single-arm zero events and double-arm zero events, which may interfere with the statistical inference of the effect size and complicate the calculation. However, if the studies of double-arm zero events are excluded from the meta-analysis, the estimation of the overall effect size may be biased (34). This study did not include double-arm zero events, which may have a certain impact on the results. Another limitation is that we only classified and compared the drug categories of the treatment group and the control group, and did not identify the drugs in detail, which may affect the accuracy of the result analysis.

## Conclusions

Our study has shown that different ICI treatment regimens show differents in the risk of cardiotoxicity. For ICI monotherapy, CTLA-4 may be linked to higher levels of cardiotoxicity than PD-1 or PD-L1. For dual therapy, the risk of cardiotoxicity associated with dual ICI therapy was higher than that with single chemotherapy or single targeted therapy. This study also gives evidence for the incidence and risk ratio of ICI-related cardiotoxicity and provide reference for individualized treatment of cancer patients.

## Author contributions

Concept and Design: JY, XC and ML. Collection and assembly of data: XC and RN. Data analysis and interpretation: JY, XC, RN and BZ. Manuscript writing: ML, JY and XC. Manuscript revision: ML and SH. All authors contributed to the article and approved the submitted version.

## Funding

This work was supported by The Startup Fund for Scientific Research, Fujian Undergraduate Education and teaching reform research Major project (Grant number: FBJG20210119), Fujian Provincial health technology project (Grant number: Fujian Provincial health technology project) and Natural Science Foundation of Fujian Province (Grant number: 2021J011304). The Joint Fund for the Innovation of Science and Technology, Fujian Province (Grant number 2018Y9037).

## Conflict of interest

The authors declare that the research was conducted in the absence of any commercial or financial relationships that could be construed as a potential conflict of interest.

## Publisher’s note

All claims expressed in this article are solely those of the authors and do not necessarily represent those of their affiliated organizations, or those of the publisher, the editors and the reviewers. Any product that may be evaluated in this article, or claim that may be made by its manufacturer, is not guaranteed or endorsed by the publisher.

## Abbreviations

Chemo, chemotherapy; CTLA-4, anti-cytotoxic T-lymphocyte antigen-4; CTCAEs, common terminology criteria for adverse events; CI(s), confidence interval(s); ICI(s), immune checkpoint inhibitor(s); irAEs, immune-related adverse events; PD-1, anti-programmed cell death 1; PD-L1, anti-programmed cell death ligand 1; RCTs, randomized clinical trials; RR(s), relative risk(s); SUCRA, surface under the cumulative ranking; TTD, targeted therapy drug.
