# Supplementary material for: Cardiotoxicity of immune checkpoint inhibitors: A frequency network meta-analysis
Source: Front Immunol. 2022 Sep 14;13:1006860. doi: 10.3389/fimmu.2022.1006860 (PMC9515416; doi:10.3389/fimmu.2022.1006860)
Supplement: Supplementary file 1 [file DataSheet_1.docx]

Supplementary Material

# Supplementary Figures and Tables

## Supplementary Figures

**(A)**

**(B)**

**(C)**

**(D)**

**Supplementary Figure 1.** Begg’s funnel plot of caridiotoxicity. (A): Begg’s funnel plot of Grade 1-5 caridiotoxicity, (B): Begg’s funnel plot of Grade 3-5 caridiotoxicity, (C): Begg’s funnel plot of Grade 1-5 myocarditis, (D): Begg’s funnel plot of Grade 3-5 myocarditis.


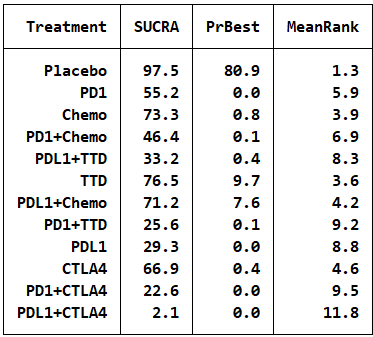

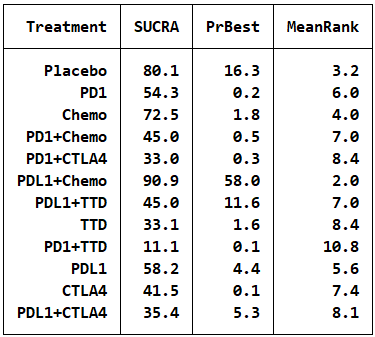

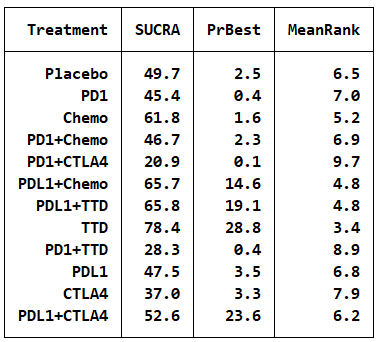

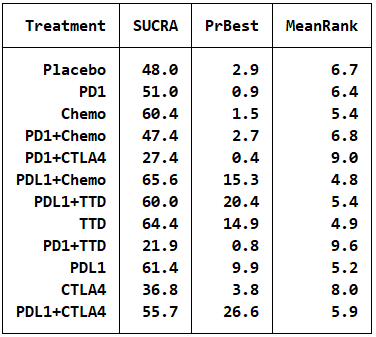


**(A)**

**(B)**

**(C)**

**(D)**

**Supplementary Figure 2.** Relative ranking of different treatments. (A): Grade 1-5 caridiotoxicity, (B): Grade 3-5 caridiotoxicity, (C): Grade 1-5 myocarditis, (D): Grade 3-5 myocarditis.

**(A)**

**(B)**

**(C)**

**(D)**

**Supplementary Figure 3.** Subgroup analysis according to does. (A): Atezolizumab + TTD vs. TTD, (B): Nivolumab vs. Chemo, (C): Nivolumab vs. Placebo, (D): D: Durvalumab vs. Chemo. Abbreviations: Atezo: Atezolizumab, Niv: Nivolumab, Dur: Durvalumab.


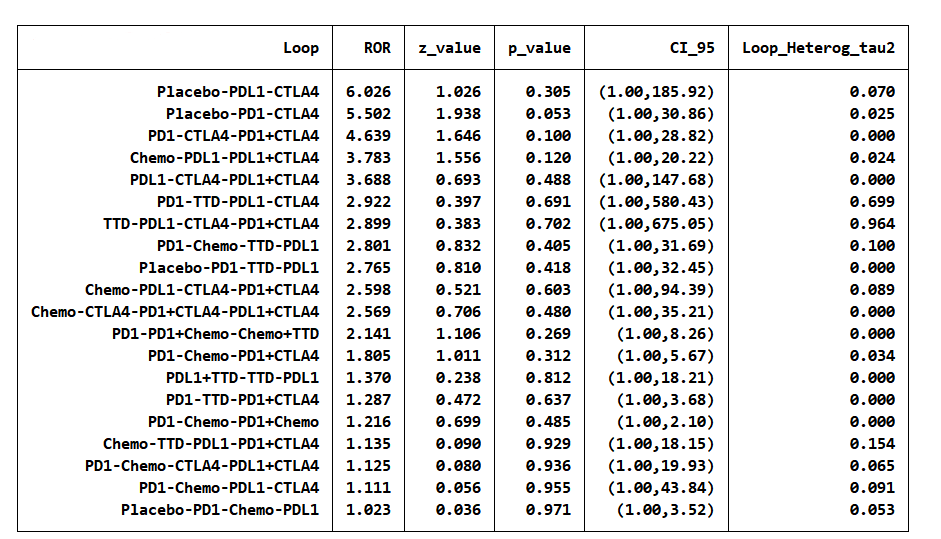

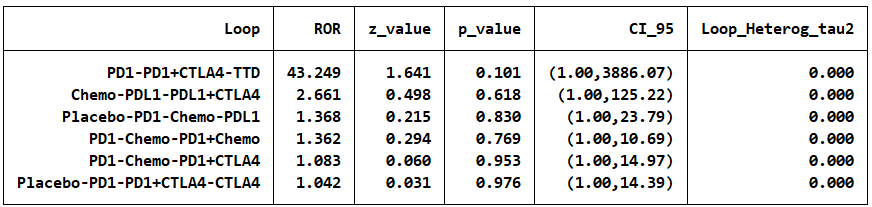

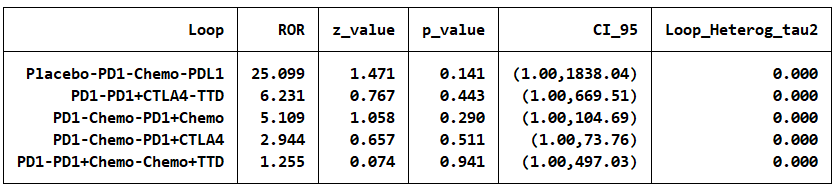

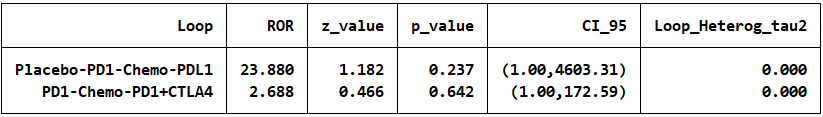


(A)

(B)

(C)

(D)

**Supplementary Figure 4.** Ring inconsistency test results. (A): Grade 1-5 caridiotoxicity, (B): Grade 3-5 caridiotoxicity, (C): Grade 1-5 myocarditis, (D): Grade 3-5 myocarditis.

## Supplementary Tables

**Supplementary Table 1.** Search strategy in PubMed, Embase, Web of Science and Cochrane Library.

|  | **Search strategy in PubMed** |
| --- | --- |
| P | #1  “Neoplasms”[mh] OR Neoplasms[tiab] OR Neoplasm[tiab] OR Cancer[tiab] OR Cancers[tiab] OR Tumors [tiab] OR Tumor[tiab] OR Malignancy[tiab] OR Malignancies[tiab] |
| I | #2  Programmed death ligand 1[tiab] OR PD-L1[tiab] OR Programmed death 1[tiab] OR PD-1[tiab] OR Cytotoxic T-lymphocyte antigen 4[tiab] OR CTLA-4[tiab] OR Immune Checkpoint Inhibitor[tiab] OR immune therapy[tiab] OR immunotherapy[tiab] OR Nivolumab[tiab] OR MDX-1106[tiab] OR ONO-4538[tiab] OR BMS-936558[tiab] OR Opdivo [tiab] OR Pembrolizumab[tiab] OR lambrolizumab[tiab] OR Keytruda[tiab] OR MK-3475[tiab] OR Cemiplimab[tiab] OR Toripalimab[tiab] OR JS001[tiab]OR Sintilimab[tiab] OR Durvalumab [tiab] OR Atezolizumab[tiab] OR MPDL3280A[tiab] OR tecentriq[tiab] OR RG7446[tiab] OR RG-7446[tiab] OR Avelumab[tiab] OR MSB0010718C[tiab] OR Ipilimumab[tiab] OR Anti CTLA 4 MAb Ipilimumab[tiab] OR Anti-CTLA-4 MAb Ipilimumab[tiab] OR Ipilimumab, Anti-CTLA-4 MAb[tiab] OR Yervoy[tiab] OR MDX010[tiab] OR MDX 010[tiab] OR MDX-010 M[tiab] OR MDX CTLA 4[tiab] OR Tremelimumab[tiab] OR ticilimumab[tiab] OR CP 675[Title/Abstract] OR CP675 cpd[tiab] OR CP-675[tiab] OR CP-675,206[tiab] OR CP-675206[tiab] OR CP675206[tiab] OR CP 675206[tiab]ORCamrelizumab[tiab]OR SHR-1210[tiab]OR SHR 1210[tiab] |
| S | #3  (randomized controlled trial[pt] OR controlled clinical trial[pt] OR randomized[tiab] OR placebo[tiab] OR clinical trials as topic[mesh:noexp] OR randomly[tiab] OR trial[ti]) NOT (animals[mh] NOT (humans[mh] AND animals[mh])) |
|  | #4"2014/01/01"[Date - Publication] : "2022/1/1"[Date - Publication] |
|  | #5  #1AND#2AND#3 |
|  | **Search strategy in Embase** |
| P | #1  'neoplasms':ti,ab,kw OR 'neoplasm':ti,ab,kw OR 'cancer':ti,ab,kw OR 'cancers':ti,ab,kw OR 'tumors':ti,ab,kw OR 'tumor':ti,ab,kw OR 'malignancy':ti,ab,kw OR 'malignancies':ti,ab,kw OR 'neoplasms'/exp |
| I | #2  'programmed death ligand 1':ti,ab,kw OR 'pd-l1':ti,ab,kw OR 'programmed death 1':ti,ab,kw OR 'pd-1':ti,ab,kw OR 'cytotoxic t-lymphocyte antigen 4':ti,ab,kw OR 'ctla 4':ti,ab,kw OR 'immune checkpoint inhibitor':ti,ab,kw OR 'immune therapy':ti,ab,kw OR 'immunotherapy':ti,ab,kw OR 'nivolumab':ti,ab,kw OR 'mdx-1106':ti,ab,kw OR 'ono-4538':ti,ab,kw OR 'bms-936558':ti,ab,kw OR 'opdivo':ti,ab,kw OR 'pembrolizumab':ti,ab,kw OR 'lambrolizumab':ti,ab,kw OR 'keytruda':ti,ab,kw OR 'mk-3475':ti,ab,kw OR 'cemiplimab':ti,ab,kw OR 'toripalimab':ti,ab,kw OR 'JS001':ti,ab,kw OR 'sintilimab':ti,ab,kw OR 'durvalumab':ti,ab,kw OR 'atezolizumab':ti,ab,kw OR 'mpdl3280a':ti,ab,kw OR 'tecentriq':ti,ab,kw OR 'rg7446':ti,ab,kw OR 'rg-7446':ti,ab,kw OR 'avelumab':ti,ab,kw OR 'msb0010718c':ti,ab,kw OR 'ipilimumab':ti,ab,kw OR 'anti ctla 4 mab ipilimumab':ti,ab,kw OR 'anti-ctla-4 mab ipilimumab':ti,ab,kw OR 'ipilimumab, anti-ctla-4 mab':ti,ab,kw OR 'yervoy':ti,ab,kw OR 'mdx010':ti,ab,kw OR 'mdx 010':ti,ab,kw OR 'mdx-010 m':ti,ab,kw OR 'mdx ctla 4':ti,ab,kw OR 'tremelimumab':ti,ab,kw OR 'ticilimumab':ti,ab,kw OR 'cp 675':ti,ab,kw OR 'cp675 cpd':ti,ab,kw OR 'cp-675':ti,ab,kw OR 'cp-675,206':ti,ab,kw OR 'cp-675206':ti,ab,kw OR 'cp675206':ti,ab,kw OR 'cp 675206':ti,ab,kw OR 'Camrelizumab':ti,ab,kw OR 'SHR-1210':ti,ab,kw OR 'SHR 1210':ti,ab,kw |
| S | #3  'crossover procedure':de OR 'double-blind procedure':de OR 'randomized controlled trial':de OR 'single-blind procedure':de OR (random* OR factorial* OR crossover* OR cross NEXT/1 over* OR placebo* OR doubl* NEAR/1 blind* OR singl* NEAR/1 blind* OR assign* OR allocat* OR volunteer*):de,ab,ti |
|  | #4  #1 AND #2 AND #3 AND [2014-2022]/py |
|  | **Search strategy in Web of Science** |
| P | #1  TS=(Neoplasm OR Cancer OR Tumor OR Malignancy) |
| I | #2  TS=('programmed death ligand 1' OR 'programmed death 1' OR 'cytotoxic t-lymphocyte antigen 4' OR 'immune checkpoint inhibitor' OR 'nivolumab' OR 'pembrolizumab' OR 'lambrolizumab' OR 'keytruda' OR 'cemiplimab' OR 'toripalimab' OR 'sintilimab' OR 'durvalumab' OR 'atezolizumab' OR 'avelumab' OR 'ipilimumab' OR 'tremelimumab' OR 'tocilizumab' OR 'Camrelizumab' ) |
| S | #3  TS=("randomized controlled trial" OR "controlled clinical trial" OR "clinical trial" OR "random*" OR "rct*" OR "crossover" OR "masked” OR “blind*" OR "placebo*") |
|  | #4  DOP=(2014-01-01/2022-1-30) |
|  | #5  #1AND#2AND#3AND#4 |
|  | **Search strategy in Cochrane Library** |
| P | #1  MeSH descriptor: [Neoplasms] explode all trees |
|  | #2  (Neoplasms OR Neoplasm OR Cancer OR Cancers OR Tumors OR Tumor OR Malignancy OR Malignancies):ti,ab,kw |
|  | #3  #1OR#2 |
| I | #4  (“Programmed death ligand 1” OR PD-L1 OR “Programmed death 1” OR PD-1 OR “Cytotoxic T-lymphocyte antigen 4” OR CTLA-4 OR “Immune Checkpoint Inhibitor” OR “immune therapy” OR immunotherapy OR Nivolumab OR MDX-1106 OR ONO-4538 OR BMS-936558 OR Opdivo OR Pembrolizumab OR lambrolizumab OR Keytruda OR MK-3475 OR Cemiplimab OR Toripalimab OR JS001 OR Sintilimab OR Durvalumab OR Atezolizumab OR MPDL3280A OR tecentriq OR RG7446 OR RG-7446 OR Avelumab OR MSB0010718C OR Ipilimumab OR “Anti CTLA 4 MAb Ipilimumab” OR “Anti-CTLA-4 MAb Ipilimumab” OR “Ipilimumab, Anti-CTLA-4 MAb” OR Yervoy OR MDX010 OR “MDX 010” OR “MDX-010 M” OR “MDX CTLA 4” OR Tremelimumab OR ticilimumab OR “CP 675” OR “CP675 cpd” OR CP-675 OR “CP-675,206” OR CP-675206 OR CP675206 OR “CP 675206” OR Camrelizumab OR SHR-1210 OR “SHR 1210”):ti,ab,kw |
|  | #5  #3 #3 AND #4 with Publication Year from 2014 to 2022, in Trials |

**Supplementary Table 2.** Characteristics of the studies included in the meta-analysis. Abbreviations: Atezo: Atezolizumab, Ave: Avelumab, Cam: Camrelizumab, Chemo: Chemotherapy, Dur: Durvalumab, Ipi: Ipilimumab, Niv: Nivolumab, Pem: pembrolizumab, Sinti: Sintilimab, Treme: Tremelimumab, Toripa: Toripalilimab.

| First author | year | study | phase | NCT | line | Tumour type | Follow-up (month) | treatments |
| --- | --- | --- | --- | --- | --- | --- | --- | --- |
| Winer EP | 2021 | KEYNOTE-119 | 3 | NCT02555657 | ≥2 | Breast cancer | 31 | Pem vs. Chemo |
| Mok TSK | 2019 | KEYNOTE-042 | 3 | NCT02220894 | 1 | Lung | 12.8 | Pem vs. Chemo |
| Paz-Ares L | 2018 | KEYNOTE-407 | 3 | NCT02775435 | 1 | Lung | 14.3 | Pem + Chemo vs. Placebo +Chemo |
| Finn RS | 2019 | KEYNOTE-240 | 3 | NCT02702401 | 2 | Hepatocellular Carcinoma | 13.8 (pem) 10 (placebo) | Pem vs. Placebo |
| Wu YL | 2019 | CheckMate-078 | 3 | NCT02613507 | ≥2 | Lung | 10.4 (Niv) 8.8 (docetaxel) | Niv vs. docetaxel |
| Socinsk MA | 2018 | IMpower150 | 3 | NCT02366143 | 1 | Lung | 15.4 (Atezo) 15.5 (Chemo) | Atezo + bevacizumab + carboplatin + paclitaxel vs. bevacizumab + carboplatin + paclitaxel |
| Gadgeel S | 2020 | KEYNOTE-189 | 3 | NCT02578680 | 1 | Lung | 24 | Pem + pemetrexed + platinum vs. Placebo + pemetrexed +platinum |
| Bellmunt J | 2017 | KEYNOTE-045 | 3 | NCT02256436 | ≥2 | Urothelial cancer | 14.1 | Pem vs. Chemo |
| Paz-Ares L | 2019 | CASPIAN | 3 | NCT03043872 | 1 | Lung | NR | Dur + platinum-etoposide vs. platinum-etoposide |
| Motzer RJ | 2019 | JAVELIN Renal 101 | 3 | NCT02684006 | 1 | Kidney | 11.6 (Ave) 10.7 (sunitinib) | Ave +axitinib vs. sunitinib |
| Motzer R | 2021 | CLEAR | 3 | NCT02811861 | 1 | Kidney | NR | Lenvatinib + Pem vs. Lenvatinib + Everolimus vs. Sunitinib |
| Miles D | 2021 | IMpassion131 | 3 | NCT03125902 | 1 | Breast | 14.2 | Atezo + paclitaxel vs. Placebo+ paclitaxel |
| Robert C | 2015 | CheckMate 066 | 3 | NCT01721772 | 1 | Melanoma | 16.7 | Niv vs. dacarbazine |
| Lange CJ | 2016 | KEYNOTE-021 | 2 | NCT02039674 | 1 | NSCLC | 10.6 | Pem + Chemo vs. Chemo |
| Kojima T | 2020 | KEYNOTE-181 | 3 | NCT02564263 | ≥2 | Esophageal Cancer | 7.1 (Pem) 6.9 (Chemo) | Pem vs. Chemo |
| Herbst RS | 2020 | IMpower110 | 3 | NCT02409342 | 1 | NSCLC | 20.2 (Atezo) 13.1 (Chemo) | Atezo vs. Chemo |
| Herbst RS | 2016 | KEYNOTE-010 | 3 | NCT01905657 | ≥2 | NSCLC | 13.1 | Pem2mg/kg vs. Pem10mg/kg vs. docetaxe |
| Kwon ED | 2014 | CA184-043 | 3 | NCT00861614 | ≥2 | Prostate cancer | NR | Ipi vs. Placebo |
| Fennell DA | 2021 | CONFIRM | 3 | NCT03063450 | ≥2 | Mesothelioma | 11.6 | Niv vs. Placebo |
| Antonia SJ | 2017 | PACIFIC | 3 | NCT02125461 | Early (adjuvant) | Lung | NR | Dur vs. Placebo |
| Schmid P | 2020 | IMpassion130 | 3 | NCT02425891 | 1 | Breast | 18.5 (Atezo) 17.5 (placebo) | Atezo + nab-paclitaxel vs. Placebo + nab-paclitaxel |
| Eggermont AMM | 2016 | EORTC 18071 | 3 | NCT00636168 | Early (adjuvant) | Melanoma | 63.6 | Ipi vs. Placebo |
| Schmid P | 2020 | KEYNOTE-522 | 3 | NCT03036488 | Early (adjuvant) | Breast | 15.5 | Pem + Chemo vs. Placebo +Chemo |
| L. Horn | 2018 | IMpower133 | 3 | NCT02763579 | 1 | Lung | 13.9 | Atezo vs. Placebo |
| Barlesi F | 2018 | JAVELIN Lung 200 | 3 | NCT02395172 | ≥2 | Lung | 18.3 | Ave vs. docetaxel |
| Ferris RL | 2018 | CheckMate 141 | 3 | NCT02105636 | ≥2 | Head and Neck | 24.2 | Niv vs. Chemo |
| Eggermont AMM | 2018 | KEYNOTE-054 | 3 | NCT02362594 | Early (adjuvant) | Melanoma | 15 | Pem vs. placebo |
| Kang YK | 2017 | ONO-4538-12, ATTRACTION-2 | 3 | NCT02267343 | ≥2 | Gastroesophageal cancer | 8.9 (Niv) 8.6 (Placebo) | Niv vs. Placebo |
| Shitara K | 2020 | KEYNOTE-062 | 3 | NCT02494583 | 1 | Gastric Cancer | 29.4 | Pem vs. Pem + Chemo vs. Chemo |
| Beer TM | 2017 | CA184-095 | 3 | NCT01057810 | 1 | Prostate Cancer | NR | Ipi vs. Placebo |
| Fehrenbache L | 2016 | POPLAR | 2 | NCT01903993 | ≥2 | Lung | 14.8 (Atezo) 15.7 (docetaxel) | Atezo vs. docetaxel |
| Emens L | 2019 | KATE2 | 2 | NCT02924883 | ≥2 | Breast | 19.0 (Atezo) | Trastuzumab emtansine + Atezo vs. trastuzumab emtansine + placebo |
| West H | 2019 | IMpower130 | 3 | NCT02367781 | 1 | Lung | 18.5 (Atezo) 19.2 (Chemo) | Atezo + chemo vs. Chemo |
| Finn RS | 2020 | IMbrave-150 | 3 | NCT03434379 | 1 | Hepatocellular carcinoma | 8.6 | Atezo + bevacizumab vs. sorafenib |
| Loibl S | 2019 | Gepar Nuevo | 2 | NCT0268509 | Early (adjuvant) | Breast | NR | Dur vs. Placebo |
| Tolaney SM | 2020 | NR | 2 | NCT03051659 | ≥2 | Breast | 10.9 | Eribulin + Pem vs. Eribulin |
| Borghaei H | 2015 | CheckMate-057 | 3 | NCT01673867 | ≥2 | Lung | NR | Niv vs. docetaxe |
| Carbone, | 2017 | CheckMate-026 | 3 | NCT02041533 | 1 | Lung | 13.5 | Niv vs. Chemo |
| Cohen EEW | 2019 | KEYNOTE-040 | 3 | NCT02252042 | ≥2 | Head and neck | 7.5 | Pem vs. methotrexate + docetaxelor cetuximab |
| Bang YJ | 2018 | JAVELIN Gastric 300 | 3 | NCT02625623 | ≥2 | Gastric or gastroesophageal junction | 10.6 | Ave vs. Chemo |
| Jotte R | 2020 | IMpower131 | 3 | NCT02367794 | 1 | Lung | NR | Atezo + carboplatin + paclitaxel vs. Atezo + carboplatin + nab-paclitaxe vs. carboplatin + nab-paclitaxe |
| Spigel DR | 2021 | CheckMate-331 | 3 | NCT02481830 | 2 | Lung | 7.0 (Niv) 7.6 (Chemo) | Niv vs. chemo |
| Powles T | 2020 | JAVELIN Bladder 100 | 3 | NCT02603432 | 1 | Urothelial Carcinoma | NR | Ave vs. Chemo |
| Choueiri TK | 2021 | KEYNOTE-564 | 3 | NCT03142334 | Early (adjuvant) | Renal Cell Carcinoma | 24 | Pem vs. Placebo |
| Rudin CM | 2020 | KEYNOTE-604 | 3 | NCT03066778 | 1 | Lung | NR | Pem + Ep vs. placebo + Ep |
| Gutzmer R | 2020 | IMspire150 | 3 | NCT02908672 | 1 | Melanoma | 18.9 | Atezo+ vemurafenib+ cobimetinib vs. Placebo + vemurafenib + cobimetinib |
| Usmani SZ | 2019 | KEYNOTE-185 | 3 | NCT02579863 | 1 | Myeloma | 6.6 | Pem + lenalidomide + dexamethasone vs. Lenalidomide +dexamethasone |
| Powles T | 2020 | KEYNOTE-426 | 3 | NCT02853331 | 1 | Renal cell carcinoma | NR | Pem+ axitinib vs. Sunitinib |
| Powles T | 2021 | KEYNOTE-361 | 3 | NCT02853305 | 1 | Urothelial carcinoma | 31.7 | Pem+ Chemo vs. Pem vs Chemo |
| Mateos MV | 2019 | KEYNOTE-183 | 3 | NCT02576977 | ≥2 | Myeloma | 8.1 | Pem + pomalidomide + dexamethasone vs. Pomalidomide + dexamethasone |
| Maio M | 2017 | DETERMINE | 2 | NCT01843374 | ≥2 | Mesothelioma | NR | Treme vs. Placebo |
| Kuruvilla J | 2021 | KEYNOTE-204 | 3 | NCT02684292 | 1 | Hodgkin lymphoma | NR | Pem vs. brentuximab vedotin |
| Nishio M | 2021 | IMpower132 | 3 | NCT02657434 | 1 | Lung | 14.8 | Atezo + carboplatin or cisplatin + pemetrexed vs. cisplatin + pemetrexed |
| Yang Y | 2020 | NR | 3 | NCT03607539 | 1 | Lung | 8.9 | Sinti + pemetrexed + platinum vs. Placebo + pemetrexed + platinum |
| Moore KN | 2021 | IMagyn050 | 3 | NCT03038100 | 1 | Ovarian Cancer | NR | Placebo + carboplatin + paclitaxel + Bevacizumab vs. Atezo + carboplatin + paclitaxel + Bevacizumab |
| Kelly RJ | 2021 | CheckMate 577 | 3 | NCT02743494 | Early (adjuvant) | Esophageal Cancer | 24.4 | Niv vs. Placebo |
| Huang J | 2020 | ESCORT | 3 | NCT03099382 | 2 | Oesophageal carcinoma | 8.3 (Cam)  6.2（Chemo） | Cam vs. Chemo |
| Yang Y | 2021 | CAPTAIN-1st | 3 | NCT03707509 | 1 | Nasopharyngeal carcinoma | NR | Cam +gemcitabine +cisplatin vs. Placebo +gemcitabine+ cisplatin |
| Nanda R | 2020 | I-SPY2 | 2 | NCT01042379 | Early (adjuvant) | Breast | 33.6 (Pem)  42 (Chemo) | Pem + Chemo vs. Chemo |
| Mittendorf EA | 2020 | Impassion-031 | 3 | NCT03197935 | Early (adjuvant) | breast cancer | 20.6 (Atezo)  19.8 (Placebo) | Atezo + Chemo vs. Placebo + Chemo |
| Colombo N | 2021 | KEYNOTE-826 | 3 | NCT03635567 | 1 | Cervical Cancer | NR | Pem vs. Placebo |
| Rini BI | 2019 | IMmotion151 | 3 | NCT02420821 | 1 | Renal carcinoma | 12 | Atezo + bevacizumab vs. sunitinib |
| Mai HQ | 2021 | JUPITER-02 | 3 | NCT0358176 | 1 | Nasopharyngeal carcinoma | NR | Toripa + Chemo vs. Placebo + Chemo |
| Burtness B | 2019 | KEYNOTE-048 | 3 | NCT02358031 | 1 | Head and neck | 13 (Pem)  11.5 (Pem+Chemo）10.7 (Chemo) | Pem vs. pem + platinum + 5-fluorouracil vs. cetuximab + platinum + 5-fluorouracil |
| Reck M | 2019 | KEYNOTE-024 | 3 | NCT02142738 | 1 | Lung | 25.5 | Pem vs. platinum-based chemotherapy |
| Shitara K | 2018 | KEYNOTE‑061 | 3 | NCT02370498 | ≥2 | Gastric or gastroesophageal junction | 7.9 | pem vs. paclitaxel |
| Mc Dermott DF | 2018 | IMmotion150 | 2 | NR | 1 | Kidney | 20.7 | Atezo vs. Atezo + bevacizumab vs. sunitinib |
| Fehrenbacher L | 2018 | OAK | 3 | NCT02008227 | ≥2 | Lung | 28 | Atezo vs. docetaxel |
| Powles T | 2018 | IMvigor211 | 3 | NCT02302807 | ≥2 | Urothelial | 17.3 | Atezo vs. Chemo of physician’s choice (vinflunine, paclitaxel, or docetaxel) |
| Kato K | 2019 | ATTRACTION-3 | 3 | NCT02569242 | ≥2 | Oesophageal | 10.5 (Niv)  8 (Chemo) | Niv vs. investigator’s choice of chemotherapy (paclitaxel or docetaxel) |
| Govindan R | 2017 | NR | 3 | NCT01285609 | 1 | Lung | 12.5 (Ipi)  11.8 (Placebo) | Ipi +chemo vs. Placebo + Chemo |
| Larkin J | 2018 | CheckMate-037 | 3 | NCT01721746 | ≥2 | Melanoma | 24 | Niv vs. investigator’s choice chemotherapy |
| Reck M | 2016 | NR | 3 | NCT01450761 | 1 | Lung | 10.5 (Iip)  10.2 (Placebo) | Chemo + Ipi vs. Chemo + Placebo |
| Eng C | 2019 | IMblaze370 | 3 | NR | ≥2 | Colorectal | 7.3 | Atezo vs. Atezo + cobimetinib vs. regorafenib |
| Motzer RJ | 2015 | CheckMate-025 | 3 | NCT01668784 | ≥2 | Kidney | NR | Niv vs. Everolimus |
| Overman M | 2020 | KEYNOTE-144 | 2 | NCT02362048 | ≥2 | Pancreas | NR | Acalabrutinib + Pem vs. Acalabrutinib |
| Brahmer J | 2015 | CheckMate-017 | 3 | NCT01642004 | ≥2 | Lung | NR | Niv vs. docetaxel |
| Sandra P | 2018 | Alliance A091401 | 2 | NCT02500797 | ≥2 | sarcoma | NR | Niv vs. Niv+ Ipi |
| Hellmann MD | 2019 | CheckMate-227 | 3 | NCT02477826 | 1 | NSCLC | 28.3 | Niv + Ipi vs. Niv vs. Chemo |
| Rizvi NA | 2020 | MYSTIC | 3 | NCT02453282 | 1 | Lung | NR | Dur vs Dur+ Treme vs Chemo |
| Larkin J | 2019 | CheckMate-067 | 3 | NCT01844505 | 1 | Melanoma | 60 | Niv + Ipi vs. Niv vs. Ipi |
| Motzer RJ | 2018 | CheckMate-214 | 3 | NCT02231749 | 1 | Renal Carcinoma | 25.2 | Niv + Ipi vs. Sunitinib |
| Hodi FS | 2016 | CheckMate-069 | 2 | NCT01927419 | 1 | Melanoma | 24 | Niv + Ipi vs. Ipi |
| Zamarin D | 2020 | NRG GY003 | 2 | NCT02498600 | 1 | Ovarian Cancer | NR | Niv + Ipi vs. Niv |
| Baas P | 2021 | CheckMate 743 | 3 | [NCT02899299](https://clinicaltrials.gov/show/NCT02899299) | 1 | Mesothelioma | 29.7 | Niv + Ipi vs. Chemo |
| Boyer M | 2021 | KEYNOTE-598 | 3 | NCT03302234 | 1 | Lung | NR | Pem + Ipi vs. Pem +Placebo |
| Scherpereel A | 2019 | IFCT-1501 MAPS2 | 2 | NCT02716272 | ≥2 | mesothelioma | 20.1 | Niv vs. Niv + Ipi |
| Janjigian YY | 2018 | CheckMate-032 | NR | NCT01928394 | ≥2 | Esophagogastric Cancer | 28 (Niv3)  24 (Niv1+Ipi3)  22 (Niv3+Ipi1) | Niv 3mg/kg vs. Niv 1mg/kg + Ipi 3mg/kg vs. Niv 3mg/kg + Ipi 3mg/kg |
| Gettinger SN | 2021 | Lung-MAP S1400I | 3 | NCT02785952 | ≥2 | Lung | 29.5 | Niv + Ipi vs. Niv |
| Ferris RL | 2020 | EAGLE | 3 | NCT02369874 | 1 | Head and neck | 7.6 (Dur)  7.8 (Chemo) | Dur + Treme vs. Dur vs. standard of care (cetuximab, taxane, methotrexate or fluoropyrimidin) |
| Siu L | 2019 | CONDOR | 2 | NCT02319044 | ≥2 | Head and neck | NR | Dur + Treme vs. Dur vs. Treme |

**Supplementary Table 3.** Network estimates of treatment comparisons for Grade 1-5 cardiotoxicity by cancer types. The summary estimates are risk ratios (RRs) and 95% confidence intervals. (A): Breast cancer, (B): Lung cancer, (C): Urothelial carcinoma, (D): Renal carcinoma, (E): Melanoma, (F): Gastroesophageal cancer, (G): Head and neck. cancer.

**(A)**

| PD-1 |  |  |  |
| --- | --- | --- | --- |
| 8.67 (0.46,164.13) | PD-1+Chemo |  |  |
| 20.36 (0.99,420.46) | 2.35 (0.82,6.75) | PD-L1+Chemo |  |
| 10.63 (0.59,191.24) | 1.23 (0.71,2.12) | 0.52 (0.21,1.29) | Chemo |

**(B)**

| PD-1 |  |  |  |  |  |  |  |
| --- | --- | --- | --- | --- | --- | --- | --- |
| 0.70 (0.34,1.46) | PD-L1 |  |  |  |  |  |  |
| 1.04 (0.49,2.21) | 1.48 (0.63,3.47) | PD-1+Chemo |  |  |  |  |  |
| 0.89 (0.40,1.98) | 1.26 (0.52,3.09) | 0.85 (0.34,2.14) | PD-L1+Chemo |  |  |  |  |
| 0.73 (0.36,1.48) | 1.04 (0.38,2.83) | 0.70 (0.25,1.96) | 0.82 (0.28,2.37) | PD-1+CTLA-4 |  |  |  |
| 0.36 (0.11,1.20) | 0.51 (0.19,1.36) | 0.35 (0.10,1.24) | 0.41 (0.11,1.50) | 0.50 (0.12,1.97) | PD-L1+CTLA-4 |  |  |
| 1.13 (0.74,1.74) | 1.62 (0.90,2.91) | 1.09 (0.59,2.03) | 1.28 (0.65,2.52) | 1.56 (0.69,3.53) | **3.14 (1.03,9.57)** | Chemo |  |
| 1.05 (0.32,3.40) | 1.50 (0.60,3.71) | 1.01 (0.29,3.54) | 1.18 (0.33,4.27) | 1.44 (0.37,5.62) | 2.91 (0.77,10.95) | 0.93 (0.31,2.75) | Placebo |

**(C)**

| PD-1 |  |  |  |
| --- | --- | --- | --- |
| 0.49 (0.18,1.32) | PD-L1 |  |  |
| 0.85 (0.55,1.31) | 1.74 (0.64,4.72) | PD-1+Chemo |  |
| 1.13 (0.72,1.78) | 2.33 (0.95,5.71) | 1.34 (0.86,2.08) | Chemo |

**(D)**

| PD-1 |  |  |  |  |  |  |
| --- | --- | --- | --- | --- | --- | --- |
| 1.02 (0.10,10.60) | PD-L1 |  |  |  |  |  |
| 0.77 (0.18,3.35) | 0.76 (0.09,6.64) | PD-1+TTD |  |  |  |  |
| 0.44 (0.08,2.41) | 0.43 (0.08,2.34) | 0.57 (0.13,2.54) | PD-L1+TTD |  |  |  |
| 0.88 (0.17,4.49) | 0.86 (0.08,9.01) | 1.14 (0.26,4.97) | 1.99 (0.36,10.92) | PD-1+CTLA-4 |  |  |
| 1.58 (0.50,4.98) | 1.55 (0.20,11.91) | 2.04 (0.82,5.08) | **3.57 (1.03,12.44)** | 1.79 (0.56,5.70) | TTD |  |
| 2.44 (0.61,9.78) | 2.39 (0.16,36.35) | 3.15 (0.42,23.75) | 5.52 (0.62,49.36) | 2.77 (0.33,23.56) | 1.54 (0.25,9.36) | Placebo |

**(E)**

| PD-1 |  |  |  |  |
| --- | --- | --- | --- | --- |
| 0.76 (0.06,9.11) | CTLA-4 |  |  |  |
| 0.28 (0.02,4.45) | 0.37 (0.04,3.66) | PD-1+CTLA-4 |  |  |
| 1.77 (0.16,20.14) | 2.34 (0.07,74.61) | 6.26 (0.15,255.75) | Chemo |  |
| 1.92 (0.13,27.64) | 2.54 (0.22,29.33) | 6.79 (0.30,155.12) | 1.09 (0.03,37.76) | Placebo |

**(F)**

| PD-1 |  |  |  |  |  |
| --- | --- | --- | --- | --- | --- |
| 1.20 (0.14,10.22) | PD-L1 |  |  |  |  |
| 0.61 (0.21,1.76) | 0.50 (0.05,5.34) | PD-1+Chemo |  |  |  |
| 0.29 (0.01,7.07) | 0.24 (0.01,11.28) | 0.49 (0.02,13.87) | PD-1+CTLA4 |  |  |
| 1.16 (0.48,2.79) | 0.96 (0.14,6.75) | 1.91 (0.50,7.25) | 3.93 (0.15,106.55) | Chemo |  |
| 2.01 (0.72,5.57) | 1.67 (0.16,17.82) | 3.31 (0.76,14.44) | 6.81 (0.24,192.07) | 1.73 (0.45,6.67) | Placebo |

**(G)**

| PD-1 |  |  |  |  |
| --- | --- | --- | --- | --- |
| 0.79 (0.05,12.30) | PD-L1 |  |  |  |
| 0.28 (0.02,5.12) | 0.35 (0.03,3.72) | CTLA-4 |  |  |
| 0.13 (0.01,1.36) | **0.17 (0.03,0.91)** | 0.47 (0.08,2.69) | PD-L1+CTLA-4 |  |
| 0.78 (0.28,2.15) | 0.98 (0.08,12.55) | 2.77 (0.18,42.07) | 5.88 (0.72,48.14) | Chemo |
